# Traditional Chinese Medicine CFF‐1 induced cell growth inhibition, autophagy, and apoptosis via inhibiting EGFR‐related pathways in prostate cancer

**DOI:** 10.1002/cam4.1419

**Published:** 2018-03-13

**Authors:** Zhaomeng Wu, Qingyi Zhu, Yingying Yin, Dan Kang, Runyi Cao, Qian Tian, Yu Zhang, Shan Lu, Ping Liu

**Affiliations:** ^1^ Jiangsu Province Key Laboratory for Molecular and Medicine Biotechnology College of Life Sciences Nanjing Normal University Nanjing Jiangsu 210023 China; ^2^ Laboratory of Molecular Biology Jiangsu Province Hospital of TCM Nanjing Jiangsu 210029 China

**Keywords:** Prostate cancer (PCa), CFF‐1, EGFR, apoptosis, PI3K/AKT signal pathway

## Abstract

Traditional Chinese medicine (TCM) has a combined therapeutic result in cancer treatment by integrating holistic and local therapeutical effects, by which TCM can enhance the curative effect and reduce the side effect. In this study, we analyzed the effect of CFF‐1 (alcohol extract from an anticancer compound Chinese medicine) on prostate cancer (PCa) cell lines and studied in detail the mechanism of cell death induced by CFF‐1 in vitro and in vivo. From our data, we found for the first time that CFF‐1 obviously arrested cell cycle in G1 phase, decreased cell viability and then increased nuclear rupture in a dose‐dependent manner and finally resulted in apoptosis in prostate cancer cells. In molecular level, our data showed that CFF‐1 induced inhibition of EGFR auto‐phosphorylation and inactivation of EGFR. Disruption of EGFR activity in turn suppressed downstream PI3K/AKT and Raf/Erk signal pathways, resulted in the decrease of p‐FOXO1 (Ser256) and regulated the expression of apoptosis‐related and cycle‐related genes. Moreover, CFF‐1 markedly induced cell autophagy through inhibiting PI3K/AKT/mTOR pathway and then up‐regulating Beclin‐1 and LC‐3II and down‐regulating phosphorylation of p70S6K. In vivo, CFF‐1‐treated group exhibited a significant decrease in tumor volume compared with the negative control group in subcutaneous xenograft tumor in nude mice via inhibiting EGFR‐related signal pathways. Thus, bio‐functions of Chinese medicine CFF‐1 in inducing PCa cell growth inhibition, autophagy, and apoptosis suggested that CFF‐1 had the clinical potential to treat patients with prostate cancer.

## Introduction

Prostate cancer (PCa) is the second largest cancers that affect men's health in western countries. According to SEEP (Surveillance, Epidemiology, and End Results Program) statistics, there are about 220,800 new cases of prostate cancer in the United States in 2015 and 86,380 people died of prostate cancer 1. Because of the ultimately inefficiency of chemotherapy, radiation therapy, and some combination therapies, therapeutic strategies for this disease are still limited. Drug and castration resistance and metastatic disease still often develops even after potentially curative surgery 2, 3, 4. Therefore, it is of great importance to develop new therapeutics for prostate cancer treatment.

Over‐activation of EGFR has been reported in many solid tumors 5. Activation of EGFR induces phosphorylation and activation of its downstream signal pathways, such as PI3K/AKT and Raf/Erk pathways, and finally leads to cell over‐proliferation 6. Activated AKT disturbs the balance of apoptosis and cell viability by promoting NF‐*κ*B and inhibiting pro‐apoptotic transcription factor FOXOs 7, 8, 9. Activation of FOXOs (such as FoxO1 in prostate cancer cells) triggers cell cycle arrest and induces cell apoptosis via increasing the levels of Fas‐L (Fas ligand), TRAIL (tumor necrosis factor‐related apoptosis‐inducing ligand), and Bim in various types of cells 10. In addition, PI3K/AKT/mTOR/p70S6K signal pathway is the primary pathway that regulates autophagy when cells are exposed to certain conditions, such as starvation, oxidative stress, and tumor suppression. LC3‐II and Beclin‐1 play a critical role in autophagosome formation and crosstalk between autophagy and apoptosis 11, 12.

Increasing evidences suggest that occurrence of most of diseases (including cancer) is closely related to the immunosuppression, and many of traditional Chinese medicine (TCM) can correct the status of immunosuppression by improving both the immunity and the disease resistance of human bodies, and then achieve the purpose of treating diseases 13, 14. Many TCMs can enhance the immunity of human immune system when the disease is debilitating. In the complex ingredients of TCM, there are both the ingredients of holistic recuperation and the specific ingredients of targeting lesions. In cancer treatment, the specific ingredients can target cancer lesions and inhibit cancer cell growth 15. The results of clinical cancer treatment in many Hospitals of Traditional Chinese Medicine demonstrate that TCM not only has both holistic recuperation and local therapeutical effects, but also has the comprehensive effect by combining the different effects of ingredients of compound Chinese medicine. In addition, most of TCM has little cytotoxicity and side effects in the process of cancer treatment 14, 15. Although TCM has the unique advantages of whole body and complementary effect that the single medicine (western medicine) does not have in anticancer treatment 14, 15, 16, 17, the molecular mechanism of the bio‐functions of TCM in the treatment of various cancers is still not well‐known.

In this study, we investigated the effects of CFF‐1 (alcohol extract from a kind of anti‐prostate cancer TCM provided by famous TCM doctor Fusong Xu of Jiangsu Province Hospital of TCM) on the prostate cancer cell lines (including androgen‐dependent LNCaP and androgen‐independent PC3 cell lines) in vitro and in vivo. The results demonstrated for the first time that CFF‐1 could decrease the cell viability and arrest the cell cycle in the G1 phase, and further induce a significantly cell autophagy and apoptosis in prostate cancer cells. From our experimental data, CFF‐1 not only inhibited the auto‐phosphorylation of EGFR and decreased the activity of EGFR, but also impaired EGF‐enhanced auto‐phosphorylation and activation of EGFR. The inhibition degree presented a CFF‐1 dose‐dependent manner. Inhibition of EGFR activity again inhibited its downstream signal pathways (including PI3K/AKT and Raf/Erk pathways) and then decreased the activity of mTOR signal pathway and increased the activity of FOXO1. Finally, cell growth inhibition, cell autophagy, and apoptosis were induced by CFF‐1 in prostate cancer cells via down‐regulating the levels of Cyclin D1/Bcl‐2/XIAP/Survivin and up‐regulating the levels of p21/p27/Bax/FasL/Bim/c‐Caspase 3/c‐PARP‐1/LC3‐II/Beclin‐1. Thus, our experimental results here partially provided a theoretical basis to support that Chinese medicine CFF‐1 had the clinical therapeutic potential to treat patients with prostate cancer.

## Materials and Methods

### Cell culture and CFF‐1

Prostate cancer cell lines (including LNCaP, CWR22Rv1, PC3 and DU145) and normal prostate epithelial cell line (WPMY‐1) were cultured in RPMI 1640 (Wisent, Nanjing, China) supplemented with 10% FBS (BRL‐GIBCO Co. Ltd., Carlsbad, CA), penicillin (100 U/mL), and streptomycin (100 mg/mL; Sigma‐Aldrich, St. Louis, MO) in a humidified 5% CO_2_ atmosphere at 37°C.

CFF‐1 was the alcohol extract from a kind of Compound Traditional Chinese Medicine, which provided by Jiangsu Province Hospital of Traditional Chinese Medicine. It contained 1 mg/mL pharmaceutical raw materials which were composed of *Radix Aconiti Lateralis Preparata, Poria, Ramulus Cinnamomi, Radix Rehmanniae Preparta, Rhizoma Polygonati, Rhizoma Curcumae, Herba Polygoni Perfoliati, Rhizoma Wenyujin Concisum, Paris polyphylla Smith, Radix Cyathulae,* and *Radix Glycyrrhizae*, etc.

The pharmaceutical raw materials of Traditional Chinese Medicine were smashed and soaked in absolute ethanol overnight and then centrifuged and discarded the residues. The supernatant was adjusted the concentration to 1 g/ml of raw materials by low‐temperature evaporation and stocked in −80°C for the use in next experiments. In treatment of cells, CFF‐1 was used in different final concentrations of 0, 2, 5, and 10 mg/mL. To avoid the effect of ethanol on cells, the final concentration of ethanol contained in each treated culture mixture (including 0, 2, 5, and 10 mg/mL of CFF‐1) would be adjusted to the same.

### Antibody and reagents

Antibodies for PARP‐1, Caspase 3, Cyclin D1, XIAP, AKT, p‐AKT (Ser473), FOXO1, p‐FOXO1 (Ser256), Bim, p21, p27, EGFR, p‐EGFR (Tyr1173), and *β*‐actin were purchased from Santa Cruz Biotechnology (Santa Cruz, CA). Antibodies for Erk, p‐Erk1/2(T202/Y204) and p‐AKT (Ser308) were purchased from Bioworld Technology (Shanghai, China). Antibodies of p‐PI3K p85 (Tyr458) were purchased from Cell Signaling Technology (Boston, MA). Antibodies for Caspase8/p18, Caspase9/p35/p10, Bcl‐2, Bax, LC3 (‐I/‐II), Beclin‐1, mTOR, p70S6K, PI3K p85 (alpha), Survivin, p53, and Ki67 were purchased from proteintech group company (Shanghai, China). Antibodies for FasL, p‐Raf‐1(Ser338), Raf‐1, p‐MEK1/2(Ser217/Ser221), MEK1/2, p‐mTOR (Ser2448), and p‐p70S6K (Thr389/412) were purchased from Affinity Biosciences Company (Cincinnati, OH). Assay kits of RIPA, MTT, and CCK‐8 were purchased from Beyotime biotechnology (Shanghai, China). DAPI was purchased from Solarbio Science & Technology Co. Ltd. (Beijing, China) and dissolved in 1× PBS and used at a concentration of 20 *μ*g/mL. Other chemicals were all purchased from Sigma‐Aldrich Inc. (St. Louis, MO).

### DAPI staining assay for nuclear condensation rupture

For DAPI staining assay, LNCaP and PC3 cells were cultured in 12‐well plates and incubated with increasing dose of CFF‐1 (0, 2, 5, 10 mg/mL) for 24 h. Cells were washed with 1× PBS briefly and fixed in 4% formaldehyde for 15 min, and washed three times with 1× PBS and then permeabilized in 0.2% Triton X‐100 for 15 min. LNCaP and PC3 cells were then stained with DAPI (20 *μ*g/mL in 1×PBS) at room temperature for 8 min and finally were photographed by fluorescence microscopy (Nikon, IX‐71, Japan).

### Flow cytometry assay for cell apoptosis and cell cycle

LNCaP and PC3 cells were seeded in 6‐well plates and treated with different concentrations of CFF‐1 (0, 2, 5, 10 mg/mL) for 24 h; and then cells were collected for flow cytometry analysis (cell apoptosis assay) using an Annexin V‐FITC apoptosis detection kit. In detail, cells were harvested and resuspended in 1× binding buffer (contained in kit). Annexin V‐FITC and PI were added to the cells according to manufacturer's instructions for 10–20 min at room temperature. Fluorescence of the cells was read immediately by flow cytometer (Mutliskan FC; Waltham, MA, USA), and a minimum of 30,000 events was collected for each sample. Percentages of intact cells, early apoptotic cells, late apoptotic cells, and necrotic cells were directly got from dot plots and presented in bar charts.

For cell cycle assay, LNCaP and PC3 cells were seeded in 6‐well plates and treated with different concentrations of CFF‐1(0, 2, 5, 10 mg/mL) for 12 h. Cells were collected and fixed with 70% ethanol overnight at 4°C. Next day, samples were treated with 100 *μ*L of RNase A (10 mg/mL) for 30 min at 37°C, stained with 200 *μ*L of PI (10 mg/mL) for 0.5 h at 4°C, then added 800 *μ*L of 1× PBS. Fluorescence of the cells was read immediately by flow cytometer (Mutliskan FC). A minimum of 20,000 events was collected for each sample. Percentages of cells in G1 phase, S phase, and G2/M phase were directly got from dot plots and presented in bar charts.

### MTT assay and CCK‐8 assay for cell viability

MTT assay and CCK‐8 (Cell Counting Kit‐8) assay were performed to check the cell viability. Cells were seeded in a 96‐well plate at a density of 1 × 10^4^ cells/well overnight and treated with different concentrations of CFF‐1 (0, 2, 5, 10 mg/mL) for 24 h. For MTT assay, culture medium was removed and fresh medium (100 *μ*L) was added with 10 *μ*L of MTT (5 mg/mL). The plate was incubated at 37°C for 4 h in the dark. The medium was removed again, and 100 *μ*L of DMSO was added to each well. The absorbance at 570 nm was measured by a microplate reader (Thermo Scientific, Fremont, CA). For CCK‐8 assay, culture medium of cells was removed after 24 h with treatment of CFF‐1 and fresh medium (100 *μ*L) was added with CCK‐8 solution (5 *μ*L). The plate was incubated for 4 h at 37°C in the dark. Absorbance was measured using a microplate reader (Thermo Scientific, Fremont, CA) at 450 nm. The measured OD values were converted into cell viability by according to the manufacturer's protocol.

### Western blot analysis

After 24 h with the treatment of different concentrations of CFF‐1 (0, 2, 5, 10 mg/mL), LNCaP and PC3 cells were harvested and lysed with RIPA buffer (Tris–HCl, pH 7.6; 1% NP‐40; 0.1% sodium deoxycholate; 0.1% SDS; 150 mmol/L NaCl; 1 mmol/L EGTA; 1 mmol/L PMSF; 1% Triton X‐100 and Roche complete protease inhibitor cocktail). After centrifugation, supernatant was collected and total protein concentration was quantified by the Bradford Reagent (Bio‐Rad, Hercules, CA). Equal amounts of total proteins (15 *μ*g) were separated by sodium dodecyl sulfate‐polyacrylamide gel electrophoresis (SDS‐PAGE) and transferred to polyvinylidene difluoride (PVDF) membrane (Bio‐Rad Laboratories). After 1‐h blocking with 5% skim milk at room temperature, the transferred membranes were blotted using primary antibodies overnight at 4°C, and then corresponding peroxidase‐labeled secondary antibodies at room temperature for 1 h. Bands were detected using Enhanced Chemiluminescence Detection Kit (Amersham Biosciences, Danderyd, Stockholm, Sweden).

### In vivo efficacy of CFF‐1 treatment in the prostate tumor Xenograft mice model

Five‐week‐old nude mice were purchased from the Model Animal Research Center of Nanjing University, Nanjing, China. PC3 and LNCaP cells (1 × 10^6^) were suspended in PBS (100 *μ*L) and injected subcutaneously into the flanks of each animal. Mice were randomly divided into four groups (eight mice in each group), including negative control group (alcohol, the amount was same as in 5‐FU and in CFF‐1), positive control group (5‐FU, 30 mg/kg), low‐dose CFF‐1 group (0.5 g/kg), and high‐dose CFF‐1 group (2.0 g/kg); and then mice were given by intragastrical administration every 2 days for 6 weeks with negative/positive control reagents and low high doses of CFF‐1 when tumors grew to 24–30 mm^3^. The tumor length and width were measured using a caliper, and body weight was measured at the end of each treated week. The tumor volume was calculated as volume (mm^3^) = (length × width^2^)/2. At the end of experiments, mice were sacrificed on the 42nd day and tumors were dissected, weighed, and snap‐frozen for further Western‐blot analysis and immunohistochemistry analysis. All animal experiments involved in this study were approved (Permission No: NL‐129‐02) by the Ethics Committee of Jiangsu Province Hospital of TCM, Nanjing, China.

### Immunohistochemistry (IHC) analysis

Tumors were fixed in 10% buffered formalin, embedded in paraffin, sectioned at 5 *μ*m. Each tissue section was deparaffinized and rehydrated with upgraded ethanol; and then tissue sections were boiled in EDTA for 15 min, quenched with 0.3% hydrogen peroxide solution for 10 min at room temperature and blocked with BSA in PBS for 30 min. Slides were subsequently incubated with special primary antibodies as indicated in figures overnight at 4°C. Sections were counterstained with hematoxylin. Antibody binding was detected with an Envision Detection Kit, Peroxidase/DAB, Rabbit/Mouse (Gene Tech, Shanghai, China). The expression levels of special proteins were observed and photographed under a microscope at a magnification of × 400 (CTR 6000; Leica, Wetzlar, Germany), and the proliferation index was expressed as the percentage of positive cells relative to the total number of cells in a given area.

### Statistical analysis

All data were expressed as means SD. Comparison between two mean values was made by independent‐sample *t*‐test. A *P* value of <0.05 was considered to be statistically significant. All experiments were replicated three times (except for in vivo experiments).

## Results

### CFF‐1 induced morphological changes and inhibited cell viability in prostate cancer cells

**Figure 1:**
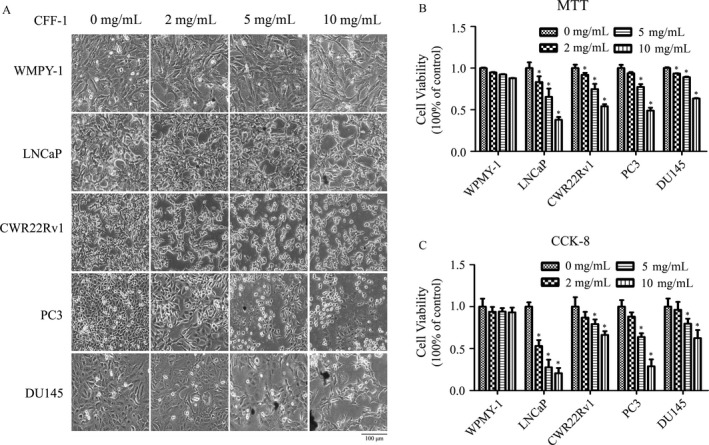
CFF‐1 induced cell morphological changes and inhibited cell viability in prostate cancer cells. (A) WPMY‐1, LNCaP, CWR22Rv1, PC3, and DU145 cells were seeded in a 12‐well plate and incubated for 24 h with different concentrations of CFF‐1 (0, 2, 5, 10 mg/mL); then, morphological changes of cells were observed and photographed by microscope (Nikon microscope, Japan) under 40× magnification. (B and C) To study proliferation and viability effects of CFF‐1, cells were treated with different concentrations of CFF‐1 (0, 2, 5, 10 mg/mL) for 24 h. The cell viability was measured using MTT and CKK‐8 assays. Experiments were carried out three times. Results are expressed as mean ± SD (*n* = 6) and as a percentage of vehicle‐treated control. *P < 0.01.

To test the effect of CFF‐1 on prostate cell lines, normal prostate epithelial cell line WPMY‐1 and prostate cancer (PCa) cell lines (including androgen‐dependent LNCaP, CWR22Rv1 and androgen‐independent PC3, DU145) were cultured and treated with CFF‐1 in different concentrations of 0, 2, 5, and 10 mg/mL for 24 h; and then the cell morphological changes were photographed by microscope and cell viabilities were determined by MTT and CCK‐8 assays. From the data, we found that CFF‐1‐induced significantly morphological changes of prostate cancer cells in a concentration‐dependent manner, such as cells, were significantly shrunken, rounded, and even some cells were burst, whereas no distinct changes on normal prostate cell WPMY‐1 even if at the treated concentration of 10 mg/mL of CFF‐1 (Fig. 1A). Moreover, MTT and CCK‐8 assays showed that the proliferation and viability of PCa cells were markedly decreased by the treatment of CFF‐1 in a concentration‐dependent manner, whereas the proliferation and viability of WPMY‐1 cells were almost not affected by the treatment of CFF‐1 (Fig. 1B and C). These results indicated that CFF‐1 not only suppressed cell growth and proliferation, but also decreased cell viability especially in prostate cancer cells.

To address the molecular mechanism of the effect of CFF‐1 on PCa cells, androgen‐dependent LNCaP and androgen‐independent PC3 cells were selected as the cell models for the next studies.

### CFF‐1 induced apoptosis and cell cycle arrest in prostate cancer cells

**Figure 2:**
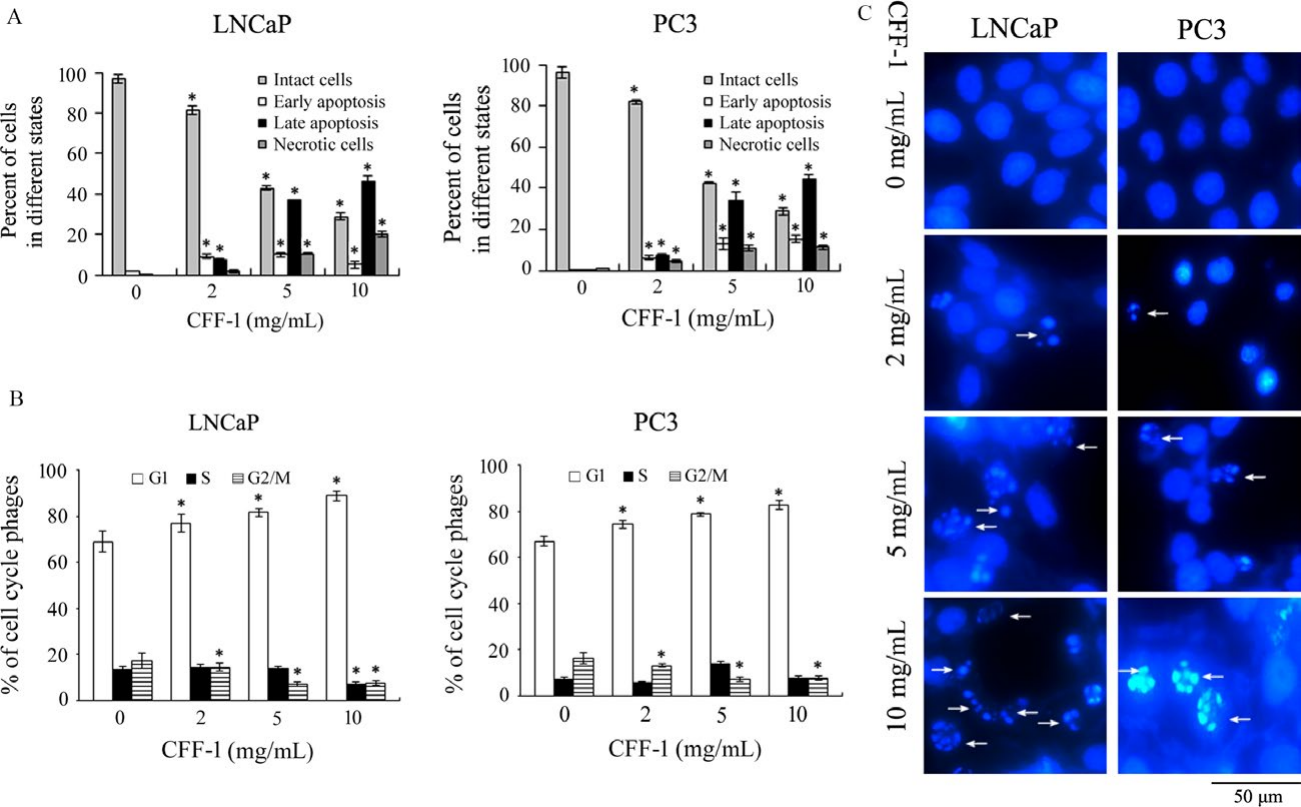
CFF‐1 induced cell cycle arrest and apoptosis in LNCaP and PC3 cells. (A) LNCaP and PC3 cells were cultured and treated with different concentrations of CFF‐1 (0, 2, 5, 10 mg/mL) for 24 h. Cells were harvested and subjected to check cell apoptosis by flow cytometric analysis. Percentages of intact cells, early apoptotic cells, late apoptotic cells, and necrotic cells were quantified. *P < 0.01. (B) LNCaP and PC3 cells were cultured in 12‐well plates overnight and then treated with different concentrations of CFF‐1 (0, 2, 5, 10 mg/mL) for 24 h. Cells were harvested and stained to analyze cell cycle by flow cytometric analysis. Percentages of cells in G1 phase, S phase, and G2/M phase were quantified. By compared to the cells treated with 0 mg/mL of CFF‐1, the CFF‐1‐induced changes of percent of cells in G1 phase, S phase, and G2/M phase were marked. *P < 0.01. (C) Cells were treated with CFF‐1 (0, 2, 5, 10 mg/mL) for 24 h, and then, the nuclear morphology was observed and photographed after DAPI staining under a microscope (Nikon microscope, Japan) using a blue filter with 40× magnification. Arrows indicate fragmented nuclei.

To identify CFF‐1‐induced cell cycle arrest and apoptosis in prostate cancer cells, LNCaP and PC3 cells were cultured and treated with different concentrations of CFF‐1 (0, 2, 5, and 10 mg/mL) for 12 h. Cells were harvested and treated for flow cytometry analysis to check cell cycle arrest and apoptosis; cells were harvested and treated for DAPI staining assay to identify PCa cell apoptosis. As shown in Figure 2A, apoptosis of PCa cells (LNCaP and PC3 cells) was induced by the treatment of CFF‐1; the degree of cell apoptosis (including early apoptosis and late apoptosis) was increased with the increasing concentration of CFF‐1. From Figure 2B, the data showed that cell cycle of LNCaP and PC3 cells was greatly arrested in G1 phase with the treatment of CFF‐1; the number of cells arrested in G1 phase was CFF‐1 concentration dependent. The number of cells in S and G2/M phases was decreased, while the number of cells in G1 phase was greatly increased with the treatment of CFF‐1. Furthermore, DAPI staining assay showed that treatment of CFF‐1‐induced formation of apoptotic bodies and nucleus shriveled in LNCaP and PC3 cells; and also, the cell numbers to form shriveled nucleus and apoptotic bodies were greatly increased with the increasing concentration of CFF‐1 (Fig. 2C, arrow pointed).

### CFF‐1 inhibited PI3K/AKT signal pathway and activated FOXO1 via down‐regulating the phosphorylation levels of PI3K, AKT, and FOXO1 in PCa cells

**Figure 3:**
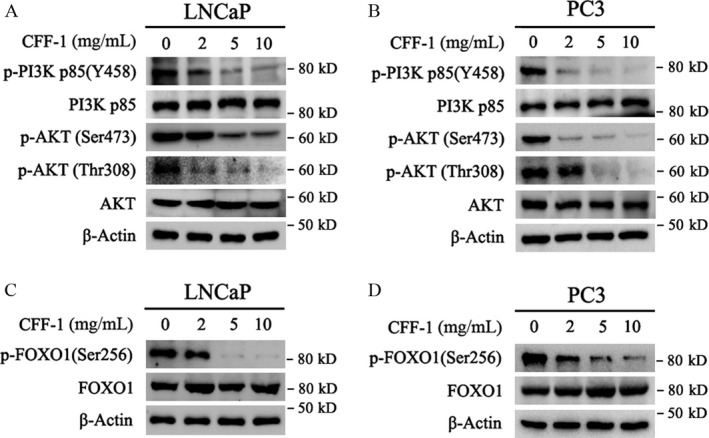
CFF‐1 inhibited PI3K/AKT signal pathway and activated FOXO1 by down‐regulating the phosphorylation levels of PI3K, AKT and FOXO1 in LNCaP and PC3 cells. (A, B, C and D) LNCaP and PC3 cells were cultured in 6‐well plates and treated with different concentrations of CFF‐1 (0, 2, 5, 10 mg/mL). After 24 h, cells were harvested and lysed for Western blot assays to check the protein levels of phospho‐PI3K (p85 subunit), PI3K (total p85), phospho‐AKT (Ser473), phospho‐AKT (Thr308), AKT, and *β*‐actin (loading control).

To explore the upstream molecular mechanisms of CFF‐1‐induced apoptosis in PCa cells, LNCaP and PC3 cells were seeded in 6‐well plates and treated with different concentrations of CFF‐1 as indicated in Figure 3. After 24 h, cells were harvested for Western blot assays. The results demonstrated that CFF‐1 treatment in PCa cells greatly decreased the levels of phospho‐AKT (p‐AKT, including Ser473 and Thr308 sites) and phospho‐PI3K (p‐PI3K) and their activities, while no obvious changes on total AKT and PI3K protein levels; and the decreasing degrees of p‐AKT and p‐PI3K levels presented in a CFF‐1 concentration‐dependent manner (Fig. 3A and B).

As we known, FOXO1 played an important role in many cellular processes, including cell growth, proliferation, and apoptosis, in prostate cancer cells; and also, FOXO1 was a key downstream target of PI3K/AKT pathway 18. It is also reported that phosphorylation of FOXO1 at Ser256 resulted in nuclear export and transcriptional inactivation of FOXO1 protein 19, 20. Therefore, we investigated the effect of CFF‐1 on FOXO1. Our data showed that phosphorylation level at Ser256, but not total protein level, of FOXO1 was decreased in LNCaP and PC3 cells with the treatment of CFF‐1; this decreasing degree was also dependent on the treated concentration of CFF‐1 (Fig. 3C and D). These results indicated that CFF‐1 could increase the transcriptional activity of FOXO1 by decreasing PI3K/AKT pathway induced the phosphorylation of FOXO1 (Ser256) and then translocating FOXO1 proteins into nuclei from cytoplasm.

### CFF‐1 induced activation of both intrinsic and extrinsic apoptotic pathways in a p53‐independent manner in PCa Cells

**Figure 4:**
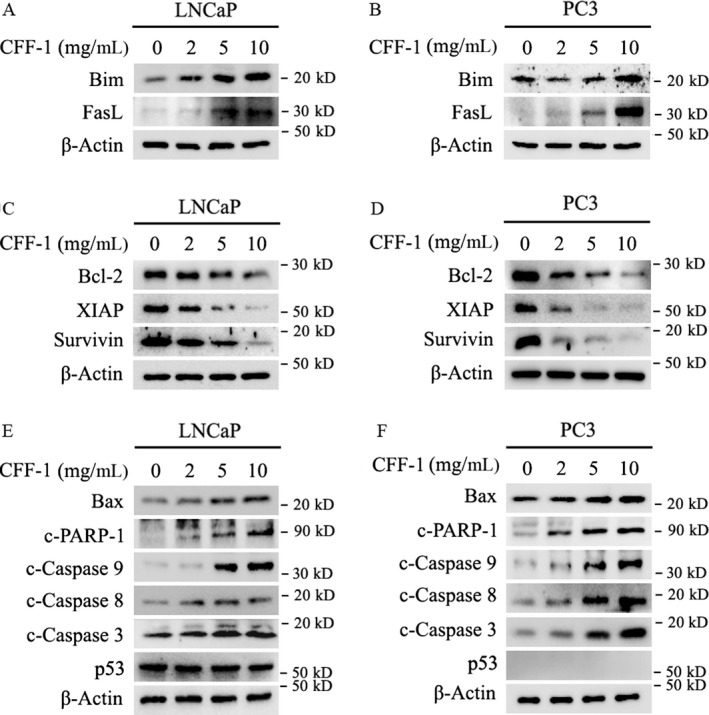
CFF‐1 induced activation of both intrinsic and extrinsic apoptotic pathways in a p53‐independent manner in LNCaP and PC3 cells. (A and B) LNCaP and PC3 cells were incubated overnight and treated with different concentrations of CFF‐1 (0, 2, 5, 10 mg/mL) for 24 h; then cells were harvested for Western blot assays to check the protein levels of Bim, Fas‐L, and *β*‐Actin (loading control). (C and D) LNCaP and PC3 cells with treatment as above were lysed for Western blot analysis to detect the protein levels of Bcl‐2, XIAP, Survivin and *β*‐Actin (loading control). (E and F) Whole cell lysates of LNCaP and PC3 cells with treatment as above were lysed and subjected to Western blot analysis to determine the protein levels of Bax, c‐Caspase‐9/‐8/‐3, c‐PARP‐1, p53, and *β*‐Actin (loading control).

It is reported that FOXO1 was a tumor suppressor and over‐activation of FOXO1 could induce cancer cell apoptosis not only by stimulating expression of death receptor ligands, like FasL (Fas ligand) and TRAIL (tumor necrosis factor‐related apoptosis‐inducing ligand), but by inducing expression of multiple pro‐apoptotic Bcl‐2 family members (mitochondria‐targeting proteins), like Bim 10. FasL can trigger the extrinsic apoptotic pathway through binding to its receptor Fas expressed on most cancer cells, while Bim can trigger the intrinsic apoptotic pathway 10. Our experimental data here showed that treatment of CFF‐1 markedly increased the expression of FOXO1‐targeted pro‐apoptotic *Bim* and *FasL* genes in a CFF‐1 concentration‐dependent manner (Fig. 4A and B). Furthermore, treatment of CFF‐1 greatly decreased the anti‐apoptotic protein levels (including Bcl‐2, XIAP and Survivin; Fig. 4C and D), while increased apoptotic proteins levels (including Bax, c‐PARP‐1, c‐Caspase9, c‐Caspase8, and c‐Caspase3; Fig. 4E and F) in LNCaP and PC3 cells. It indicated that treatment of CFF‐1 in PCa cells activated both intrinsic and extrinsic apoptotic pathways simultaneously by increasing the expression of *Bim* and *FasL* genes via activating FOXO1.

p53 is a well‐known tumor suppressor, which is known to cause cell cycle arrest, autophagy, and apoptosis in many types of cancer cells 21. In this study, we found that the protein level of p53 was almost not altered by treated CFF‐1 in LNCaP cells (no p53 expression in PC3 cells; Fig. 4E and F); the phosphorylation level of p53 at Ser15 (which is related to the activation of p53) was also down‐regulated by CFF‐1 treatment in LNCaP cells (data not shown).

Together, our data suggested that treatment of CFF‐1 inhibited PI3K/AKT pathway and activated FOXO1, and finally activated both intrinsic and extrinsic apoptosis pathways (Caspase dependent) in prostate cancer cells; the CFF‐1‐induced PCa cell apoptosis was p53‐independent.

### CFF‐1 inhibited activity of Raf/MEK/Erk signal pathway, resulting in down‐regulation of Cyclin D1 and up‐regulation of p21 and p27 in PCa cells

**Figure 5:**
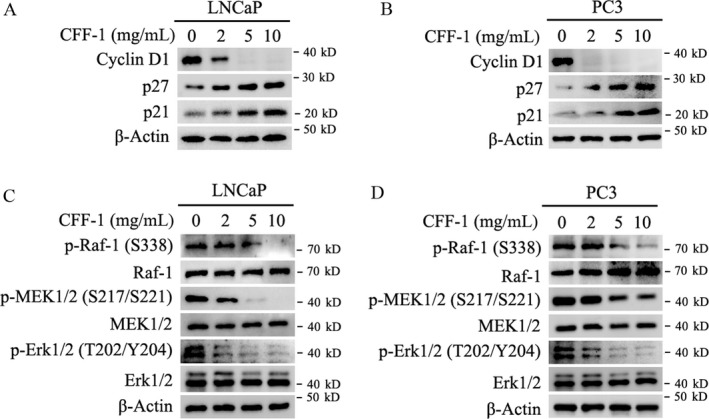
CFF‐1 inhibited the activity of Raf/MEK/Erk signal pathway, resulting in down‐regulation of Cyclin D1 and up‐regulation of p21 and p27 in PCa cells. (A and B) LNCaP and PC3 cells were cultured overnight and treated with different concentrations of CFF‐1 (0, 2, 5, 10 mg/mL) for 24 h as indicated in figures. And then cells were harvested for Western blot assays to check the protein levels of Cyclin D1, p27, p21, and *β*‐Actin (loading control). (C and D) LNCaP and PC3 cells were treated as same as A and B for 24 h. Cells were harvested and lysed for Western blot assays to check the protein levels of p‐Raf (Ser338), Raf, p‐MEK1/2 (S217/S221), MEK1/2, p‐ERK1/2 (T202/Y204), ERK1/2, and *β*‐Actin (loading control).

As we known, inhibition of Raf/MEK/Erk signal pathway down‐regulates Cyclin D1 and up‐regulates p21/p27 by activating transcription factor FOXO1, resulting in cell cycle arrest in G1 phase 10, 22. To determine that Raf/MEK/Erk signal pathway was involved in cell cycle arrest in G1 phase in CFF‐1‐treated PCa cells, LNCaP and PC3 cells were cultured and treated with different concentrations of CFF‐1 as indicated in Figure 5 for 24 h; then, cells were harvested for Western blot assay to check the protein levels of cell cycle‐related Cyclin D1, p21, and p27. As shown in Figure 5A and B, treatment of CFF‐1 in LNCaP and PC3 cells obviously decreased the protein level of Cyclin D1, whereas distinctly increased the protein levels of p21 and p27. It indicated that treatment of CFF‐1 induced cell cycle arrest in G1 phase in LNCaP and PC3 cells; and it is consistent with the results of flow cytometry analysis in Figure 2B.

In addition, treatment of CFF‐1 decreased the phosphorylation level of Raf‐1 (Ser338) in a CFF‐1 concentration‐dependent manner, while no obviously effect on the total protein level of Raf‐1 in both LNCaP and PC3 cells; inactivation of Raf‐1 further resulted in the decrease in phosphorylation levels of MEK1/2 (Ser217/Ser221) and Erk1/2 (Thr202/Tyr204), while no effect on the total protein levels of MEK1/2 and Erk1/2 in both LNCaP and PC3 cells (Fig. 5C and D). These results suggested that CFF‐1 induced cell cycle arrest and cell growth inhibition by inactivating the Raf/MEK/Erk signal pathway.

### CFF‐1 induced cell autophagy via dysregulating mTOR/p70S6K pathway in PCa cells

**Figure 6:**
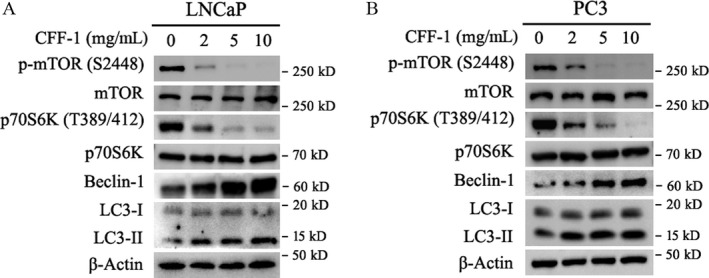
The expressions of autophagy‐related proteins in LNCaP and PC3 cells after treated with different concentrations of CFF‐1. (A and B) LNCaP and PC3 cells were incubated and treated with increasing doses of CFF‐1 (0, 2, 5, 10 mg/mL) for 24 h as indicated in figures. And then, cells were harvested and lysed for Western blot assays to check the protein levels of p‐mTOR(Ser2448), mTOR, p‐p70S6K(Thr389/412), p70S6K, Beclin‐1, LC3‐I, LC3‐II, and *β*‐Actin (loading control).

mTOR is an important factor involved in a number of cellular events and physiological process, including cell growth, cell autophagy, and homeostasis etc.; mTOR is also a downstream factor of and regulated by PI3K/AKT pathway originating from starvation, growth factors, and cellular stresses 23. To explore whether mTOR‐related cell autophagy was involved in CFF‐1‐induced cell growth inhibition and cell apoptosis, LNCaP and PC3 cells were cultured in 6‐well plates and treated with different concentrations of CFF‐1 as indicated in Figure 6 for 24 h; then, cells were harvested and lysed for Western blot assay. Our data showed that treatment of CFF‐1 in PCa cells greatly decreased phosphorylation level of mTOR (Ser2448) and resulted in mTOR activity inhibition, while no significantly effect on total protein level of mTOR (Fig. 6A and B). Next, inhibition of mTOR activity further resulted in activity inhibition of p70S6K by decreasing the phosphorylation level of p70S6K (Thr389/Thr412) in both LNCaP and PC3 cells. Finally, the levels of direct marker LC3‐II of cell autophagy were greatly elevated with the inhibition of p70S6K, although the levels of LC3‐I were not obviously changed (Fig. 6A and B). All the changes above were CFF‐1 concentration dependent as shown in figures. In addition, another important marker of cell autophagy, Beclin‐1 which negatively correlated with Bcl‐2, was also significantly increased with the treatment of CFF‐1 in a concentration‐dependent manner via decreasing the protein level of Bcl‐2 (Figs. 4C,D and 6A,B).

Combining with CFF‐1‐induced inhibition of PI3K/AKT signaling pathway (Fig. 3A and B), it is clearly that CFF‐1‐induced inhibition of mTOR/p70S6K signal pathway and cell autophagy in LNCaP and PC3 cells was via inhibiting the activity of PI3K/AKT signal pathway, suggesting cell autophagy was involved in CFF‐1‐induced cell growth inhibition and apoptosis in prostate cancer cells.

### CFF‐1 induced inhibition of auto‐phosphorylation of EGFR and inhibited activation of EGFR

**Figure 7:**
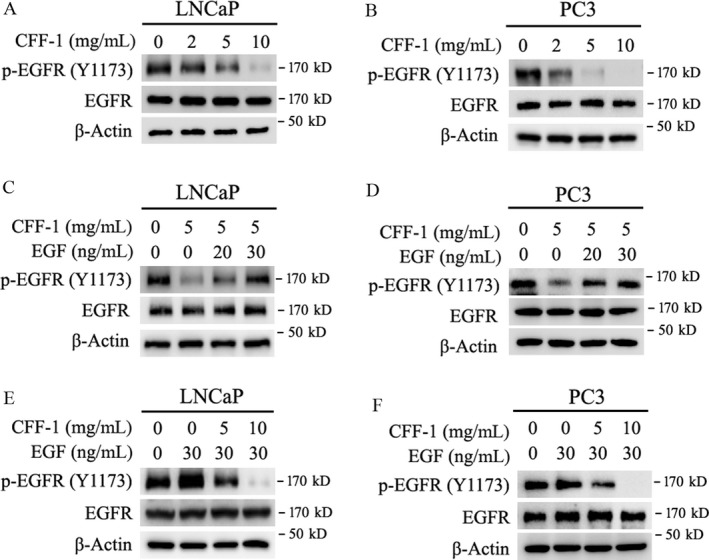
Level changes of p‐EGFR and EGFR in LNCaP and PC3 cells treated with CFF‐1 or EGF and co‐treated with CFF‐1 and EGF. (A and B) LNCaP and PC3 cells were incubated and treated with increasing doses of CFF‐1 (0, 2, 5, 10 mg/mL) for 24 h as indicated in figures. And then, cells were harvested for Western blot assays to check the protein levels of p‐EGFR (Tyr1173), EGFR, and *β*‐Actin (loading control). (C and D) LNCaP and PC3 cells were incubated and treated with 5 mg/mL of CFF‐1 and then stimulated by different concentrations of EGF (20 *μ*g/mL, 30 *μ*g/mL) for 10 min as indicated in figures. Cells were harvested and lysed for Western blot assay to check the protein levels of p‐EGFR (Tyr1173), EGFR and *β*‐Actin (loading control). (E and F) LNCaP and PC3 cells were pretreated with the indicated concentrations of CFF‐1 (0, 5, 10 mg/mL) for 24 h and then stimulated by EGF (30 *μ*g/mL) for 10 min as indicated in figures. Cells were harvested for Western blot assays to determine the levels of p‐EGFR (Tyr1173) and EGFR.

It is reported that PI3K/AKT and Raf/MEK/Erk signal pathways are two important downstream signal pathways of EGF/EGFR signal pathway 24. Over‐activation of EGF/EGFR signal pathway is often happened in many types of cancers and has been shown to increase cancer cell proliferation, enhance tumor vascularization, and promote cancer cell metastasis 25, 26. To determine that CFF‐1‐induced inhibition of PI3K/AKT and Raf/MEK/Erk signal pathways was via CFF‐1 inhibiting EGFR auto‐phosphorylation and inactivating EGF/EGFR signal pathway, LNCaP and PC3 cells were cultured and treated with different concentrations of EGF and/or CFF‐1 (5 mg/mL) or different concentrations of CFF‐1 and/or EGF (30 ng/mL) as indicated in Figure 7. From our experiment data, treatment of CFF‐1 significantly decreased auto‐phosphorylation levels of EGFR (p‐EGFR (Tyr1173)) instead of changing the total protein level of EGFR in both LNCaP and PC3 cell lines; the decreasing degree of p‐EGFR (Tyr1173) levels was dependent on the treated concentration of CFF‐1 (Fig. 7A and B).

When LNCaP and PC3 cells were treated or co‐treated with CFF‐1 and EGF, we found that CFF‐1‐induced inhibition of EGFR auto‐phosphorylation was impaired by co‐treated EGF (Fig. 7C and D). Conversely, the EGF‐induced enhancement of EGFR auto‐phosphorylation was also greatly impaired by co‐treated CFF‐1 in both LNCaP and PC3 cells (Fig. 7E and F).

Thus, our results demonstrated that CFF‐1‐induced activity inhibition of EGFR and its downstream signal pathways, including PI3K/AKT and Raf/Erk pathways, was initiated by CFF‐1‐induced inhibition of auto‐phosphorylation of EGFR.

### CFF‐1 suppressed the growth of human PCa cells in vivo by inhibiting EGFR‐related signal pathways and inducing cell autophagy and apoptosis

**Figure 8:**
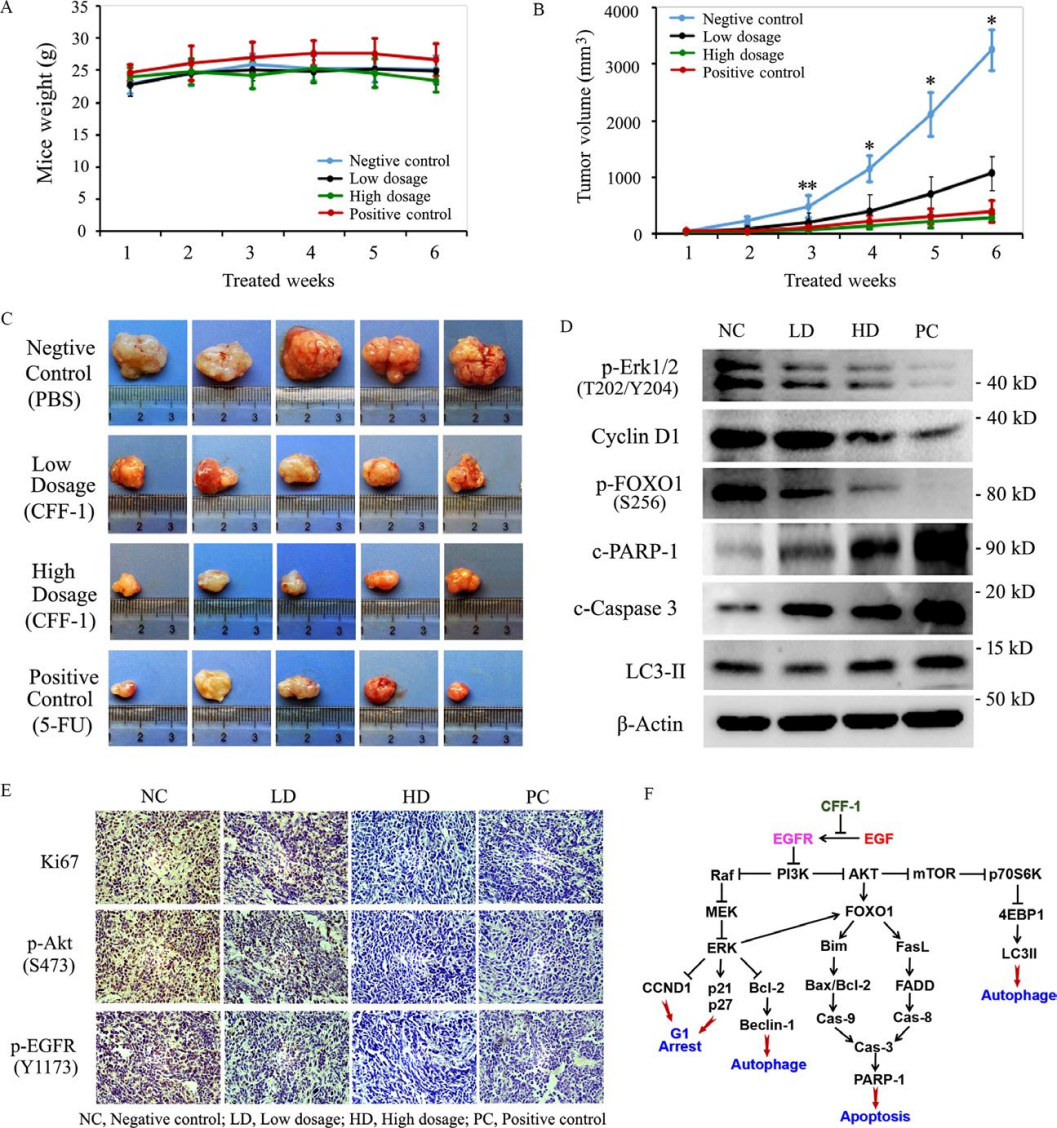
CFF‐1 suppressed tumor growth by inhibiting cell growth and inducing cell autophagy and apoptosis in human PC3 cell xenograft mice models. PC3 cell tumor xenograft nude mice were treated by oral gavage with CFF‐1 and control reagents every 2 days for 6 weeks as indicated in figures, and tumor size and mice weight were monitored at the end of each treated week. At the end of experiments, the subcutaneous tumors were resected, weighted, photographed, and measured immediately; then the tumor samples were divided and subjected to immunohistochemistry (IHC) assay and Western blot assay. (A) Average tumor volume of alcohol‐treated (*n* = 8), CFF‐1‐treated (including low‐dose and high‐dose groups, eight mice each group), and 5‐FU‐treated (*n* = 8) nude mice at the end of each treated weeks. (B) Mice weight of four groups was measured at the end of each treated weeks. *P < 0.01, **P < 0.05. (C) Tumor photographs of all groups of mice at the end of 6 weeks (presented five tumors each group). (D) Expression of proteins, such as c‐Caspase 3, c‐PARP‐1, p‐Erk1/2, Cyclin D1, LC3‐II, and p‐FOXO1, was checked by Western blot assay in tumor tissue samples. (E) IHC of Ki‐67, p‐AKT (Ser473), and p‐EGFR (Y1173) in tumor tissues. (F) Schematic diagram of signal pathways in CFF‐1‐induced cell cycle arrest in G1 phase, autophagy, and apoptosis in human prostate cancer (PCa) cells. NC, Negative Control; LD, Low dosage of CFF‐1; HD, High dosage of CFF‐1; PC, Positive Control (5‐FU).

To validate our findings from in vitro studies and test the efficacy of CFF‐1 for prostate cancer therapy, we performed in vivo study using a PC3 cell tumor xenograft model in Nude mice as described in “Methods and Materials.” In the process of intragastrical administration, tumor growth and mice weight were monitored at the end of each treated week. After treatment for 6 weeks, mice were euthanized and the subcutaneous tumors were resected, photographed, and measured immediately; then, tumors were divided and subjected to immunohistochemistry (IHC) assay and Western blot assay. By compared with negative control group, treatment of CFF‐1 showed a time‐dependent inhibition of prostate tumor growth in vivo, and apparently reduction in tumor volume was observed at second week in CFF‐1‐treated group, while no sign of toxicity was observed during the whole treatment period as depicted by a stable body weight (Fig. 8A and B). Data of Figure 8A and C also demonstrated that inhibition of tumor growth by high‐dose CFF‐1 was almost the same as that by 5‐FU (positive control). At the end of experiment, the tumor size in mice was not only obviously reduced by the treatment of CFF‐1, but the reduction was clearly presented in a CFF‐1 dose‐dependent manner (Fig. 8C). Moreover, we detected the protein levels of Ki‐67 (marker of cell proliferation), p‐EGFR (Y1173), and p‐AKT (S473) using IHC assay in PC3 tumor tissues from different groups. The results showed that treatment of CFF‐1 dramatically decreased the protein levels of Ki‐67, p‐EGFR (Y1173), and p‐AKT (S473) in tumor tissues in vivo (Fig. 8E). In addition, Western blot data of PC3 tumor tissues demonstrated that levels of p‐ERK1/2, p‐FOXO1(S256), and CyclinD1 were all down‐regulated by the treatment of CFF‐1, whereas protein levels of c‐Caspase 3, c‐PARP‐1, and LC3‐II were up‐regulated by the treatment of CFF‐1 (Fig. 8D). Therefore, in vivo experiments also implicated that treatment of CFF‐1 induced PCa cell autophagy and apoptosis by inhibiting EGFR/PI3K/AKT and EGFR/PI3K/ERK signal pathways, finally resulting in PCa cell growth inhibition.

## Discussion

Prostate cancer is one of the most common malignancies worldwide with substantial mortality and morbidity in men. Over 160,000 new patients with prostate cancer will be diagnosed each year 27, 28. Although the survival rate was greatly risen (five‐year survival rate has reached 99%, and 10‐year is over 90%) with the combination of early detection and diagnoses by improved treatment options, the prostate cancer is still the third leading cause of cancer‐related death in men in western countries. It is about 26,730 patients' dead in 2017 due to prostate cancer 28, 29.

With the further researches on cancer therapy, immunotherapy for cancer has been agreed to be the consensus of scientists 30. As a treasure of Chinese medical science, thousands of years of clinically practical experiences show that the compound Chinese traditional medicine has a unique advantage in treating cancer in clinic via increasing the body immunity to inhibit and kill cancer cells in vivo, but anyway its molecular mechanisms are still not known even to this day. CFF‐1 is a classic compound Chinese traditional medicine which has been used on clinic to treat the patients with prostate cancer in Jiangsu Hospital of Traditional Chinese Medicine for several years as a hospital prescription. In this study, we found that CFF‐1 targeted EGFR and competitively acted on EGFR with EGF and then inhibited auto‐phosphorylation and activity of EGFR thus inhibiting PI3K/AKT and Raf/Erk signal pathways by decreasing the phosphorylation levels of PI3K, AKT, Raf, and Erk in both androgen‐dependent and androgen‐independent prostate cancer cells (LNCaP and PC3 cells). Inhibition of the signal pathways finally induced cell cycle arrest in G1 phase, cell autophagy, and apoptosis by decreasing the protein levels of Cyclin D1/XIAP/Survivin, increasing the protein levels of c‐Caspase 3/c‐PARP‐1/p21/p27 and the protein levels of LC3‐II/Beclin‐1 (Fig. 8F).

It is well‐known that EGFR and its downstream signal pathways, including PI3K/AKT/mTOR pathway and Raf/MEK/Erk pathway, play key roles in many types of tissue cell tumorigenesis and tumor progress and metastasis, and are also important in regulating body immunity in suppressing T cell induced specially tumor necrosis via decreasing the expression of PD‐L1 25, 26, 31. In this study, we found for the first time that CFF‐1 targeted EGFR and competitively acted on EGFR with EGF, resulting in auto‐phosphorylation and activity inhibition of EGFR and then decreased the activities of PI3K/AKT and Raf/MEK/Erk pathways. It indicated that these blockades of EGFR and its downstream signal pathways further decreased the expression of PD‐L1 on cancer cell membranes by inhibiting the activity of mTOR signal pathway. It meant that CFF‐1 could up‐regulate body's anticancer immunity via activating T cells by suppressing PD‐L1 expression of cancer cells, suggesting that CFF‐1 might be a potential immunotherapy drug in clinic for patients with prostate cancer. Of course, there were several ligand receptors on cell membrane which related to activation of PI3K/AKT signal pathway and promoted cancer cell growth, proliferation, and metastasis 32. Therefore, it is necessary in the next study to clearly demonstrate if EGF/EGFR was the only ligand receptor which involved in CFF‐1 inhibiting PI3K/AKT signal pathway and inducing cell growth inhibition and apoptosis in PCa cells.

Phosphorylation of FOXO1 by AKT inhibits the transcriptional activity of FOXO1 and contributes to cell survival and growth 33, 34. Conversely, FOXO1 activation has been proposed to be a key for promoting cell apoptosis by stimulating the expression of death receptor ligands (FasL and TRAIL) and multiple pro‐apoptotic members of the Bcl‐2 family (Bim, Bcl‐2, BAX); and also, FOXO1 activation is a key for inducing the cell cycle arrest by up‐regulating the protein level of p27 35, 36. Here, we demonstrated that CFF‐1 decreased the phosphorylation level of FOXO1 Ser256 via inhibiting PI3K/AKT and Raf/Erk signal pathways and then induced cell cycle arrest in G1 phase and cell apoptosis via decreasing the expression of Bcl‐2/XIAP/Survivin, activating Fas‐L/Bim/Bax, and increasing the expression of p21/p27. Besides of FOXO1, FOXO3a was also the downstream target of PI3K/AKT pathway and the important transcription factor in regulating cell growth and apoptosis 34. Therefore, it needs to be further investigated if FOXO3a was also involved in CFF‐1‐induced cell growth inhibition and apoptosis in PCa cells. In many publications, p53 were the key proteins in cell growth inhibition and cell apoptosis promotion 37. In our study here, we found that p53 proteins were not the key factors in CFF‐1‐induced PCa cell growth inhibition and cell apoptosis.

mTOR is a rapamycin‐sensitive serine/threonine protein kinase and plays a key role in regulating cell growth, motility, and survival. Dysregulation of mTOR signaling pathway can be observed in many cancer cells with PI3K and AKT being upstream regulators of mTOR signaling pathway 38. Activation of AKT‐mTOR pathway increases the expression of PD‐L1 and results in inactivation of anti‐tumor T cells 31. Activation of mTORC1, a major rapamycin‐sensitive mTOR complex, promotes protein synthesis in response to growth factors via increasing the phosphorylation of p70S6K and presents an important role in cell autophagy 39. From our results, CFF‐1 promoted cell autophagy by increasing levels of LC3‐II and Beclin‐1, which played essential roles in autophagosome formation 40, 41, via inhibiting the activity of PI3K/AKT/mTOR signal pathway and the phosphorylation of p70S6K. As we known, most of autophagy cases were the early phenomena and initiation of cell apoptosis in cancer cells with the treatment of drugs. Here, CFF‐1 not only induced PCa cell autophagy, but induced PCa cell apoptosis via inhibiting EGFR and its downstream PI3K/AKT and Raf/Erk pathways. Therefore, it might be entirely possible that CFF‐1‐induced PCa cell autophagy was involved in CFF‐1‐initiated PCa cell apoptosis. Of course, it still needs to further study with experiments that identified the correlations existed between CFF‐1‐induced PCa cell autophagy and apoptosis.

Conclusively, we identified for the first time that anticancer Chinese traditional medicine CFF‐1 not only had the potential of specific anticancer effect, but could induce PCa cell growth inhibition, autophagy, and apoptosis in vitro and in vivo via targeting EGFR and competitively acting on EGFR with EGF and further suppressing the activity of EGFR/PI3K/AKT and EGFR/PI3K/Raf/Erk signal pathways. Our results might partially provide molecular basis for CFF‐1 application in clinic to treat patients with prostate cancer.

## Conflict of Interest

None declared.
